# Apolipoprotein E genotype and hepatitis C, HIV and herpes simplex disease risk: a literature review

**DOI:** 10.1186/1476-511X-9-8

**Published:** 2010-01-28

**Authors:** Inga Kuhlmann, Anne Marie Minihane, Patricia Huebbe, Almut Nebel, Gerald Rimbach

**Affiliations:** 1 Institute of Human Nutrition and Food Science, Christian-Albrechts-University, Hermann-Rodewald-Strasse 6, 24098 Kiel, Germany; 2 Department of Physiology, Faculty of Medical and Health Sciences, The University of Auckland, Private Bag 92019, Mail Centre, Auckland 1142, New Zealand; 3 Institute of Clinical Molecular Biology, Christian-Albrechts-University, Schittenhelmstrasse 12, 24105 Kiel, Germany

## Abstract

Apolipoprotein E is a polymorphic and multifunctional protein with numerous roles in lipoprotein metabolism. The three common isoforms apoE2, apoE3 and apoE4 show isoform-specific functional properties including different susceptibilities to diseases. ApoE4 is an accepted risk factor for Alzheimer's disease and cardiovascular disorders. Recently, associations between apoE4 and infectious diseases have been demonstrated. This review summarises how apoE4 may be involved in the infection incidence and associated pathologies of specific infectious diseases, namely hepatitis C, human immunodeficiency virus disease and herpes simplex.

ApoE4 seems to be protective against chronic hepatitis C virus infection and retards fibrosis progression. In contrast apoE4 enhances the fusion rate of human immunodeficiency virus with target cell membranes, resulting in accelerated cell entry and faster disease progression. Its association with human immunodeficiency virus-associated dementia remains controversial. Regarding herpes simplex virus infection, apoE4 intensifies virus latency and is associated with increased oxidative damage of the central nervous system, and there is some evidence that herpes simplex virus infection in combination with the apoE4 genotype may be associated with an increased risk of Alzheimer's disease. In addition to reviewing available data from human trials, evidence derived from a variety of cell culture and animal models are considered in this review in order to provide mechanistic insights into observed association between apoE4 genotype and viral disease infection and pathology.

## Introduction

Viral diseases are a global health problem. Like many other diseases, differences in the pathological features and outcome of viral diseases exist, which are undoubtedly in part due to genetic variations in the host. Apolipoprotein E (apoE), in addition to being a central mediator of lipoprotein metabolism, has an ever increasing 'repertoire' of biological functions [1,2]. As will be reviewed here, accumulating evidence indicates that apoE genotype could be an important host genetic factor affecting infectious disease risk.

### ApoE tissue sources and functions

The mature apoE protein results from the proteolytic cleavage of a 317-amino acid precursor protein and contains 299 amino acids with a molecular weight of approximately 34 kDa [3]. ApoE is synthesised primarily by the liver. It is estimated that 20-40% of total apoE is produced by extrahepatic tissues with the brain glial cells and macrophages expressing relatively high amounts, with lesser amounts produced by the kidneys, adrenals, spleen, testis and the skin [4-7]. In the circulation apoE is mainly found in the lipoprotein bound form, present in chylomicrons (CM), very low density lipoproteins (VLDL), and high density lipoproteins (HDL), at a plasma concentration of 20-60 mg/l [7].

The most well described role of apoE to-date is as a regulator of multiple steps in lipid (cholesterol and triglyceride) and lipoprotein metabolism, including VLDL synthesis and secretion, the hydrolysis of VLDL to produce low density lipoproteins (LDL) and the receptor mediated removal of triglyceride-rich lipoprotein remnants (VLDL and CM remnants) by the liver [7,8]. In addition to the systemic transport and uptake of lipoproteins, apoE is recognised as the primary shuttle protein for lipids which either enters the brain and central nervous system (CNS) via the blood-brain barrier or is endogenously synthesised [9], and also for the removal of modified lipids for excretion [10].

ApoE regulates the delivery of cholesterol and other lipids to target cells by acting as a high affinity ligand for multiple members of the LDL receptor family including the LDL receptor (LDL-R), the LDL receptor-related protein (LRP), the scavenger receptor, the VLDL receptor, and the apoE receptor 2 [11].

In addition, apoE binds to cell surface located glycosaminoglycans such as heparin sulphate proteoglycans (HSPG) [12,13] with lipoprotein uptake facilitated by either HSPG alone or an HSPG-LRP complex [12].

### ApoE genotype

The widely studied human apoE gene is located on chromosome 19, closely linked to the apoC-I/C-II gene complex [6,14,15]. To-date 72 individual single nucleotide polymorphisms (SNPs) of the human apoE gene have been identified [16]. The two SNPs rs429358 (C/T) and rs7412 (C/T) [16] determine together the three major alleles, termed Epsilon-2, Epsilon-3 and Epsilon-4. The corresponding products of these alleles, the apoE2, apoE3 and apoE4 protein isoforms, differ only by a single amino acid at two residues. ApoE3 has cysteine at residue 112 (rs429358) and arginine at residue 158 (rs7412), while apoE2 contains cysteine and apoE4 arginine at both positions [17,18]. Additionally, two minor alleles of the gene, ε1 and ε5, exist but these are present in less than 0.1% of the population [19]. The three major alleles are responsible for three homozygous (ε2/ε2, ε3/ε3, ε4/ε4) and three heterozygous (ε2/ε3, ε2/ε4, ε3/ε4) genotypes [17,18].

The amino acid substitutions affect salt bridge formation within the proteins, which ultimately impacts on lipoprotein preference, stability of the protein and on receptor binding activities of the isoforms [2,7] as shown in table 1. Overall apoE4 has a comparable or slightly higher affinity for the LDL-R relative to the apoE3 form, with apoE2 binding with less than two percent of the affinity of apoE3 [20].

**Table 1:** ApoE isoform amino acid differences and resulting chemical and physiological changes (according to Mahley and Rall [2], Minihane *et al*. [7])

Isoform	Amino acid 112	Amino acid 158	Relative charge	Lipoprotein preference	LDL receptor binding affinity
apoE2	cysteine	cysteine	0	HDL	low
apoE3	cysteine	arginine	+1	HDL	high
apoE4	arginine	arginine	+2	VLDL, chylomicrons	high

HDL: high density lipoprotein, VLDL: very low density lipoprotein

### ApoE genotype, blood lipid levels and risk of cardiovascular disease and Alzheimer's disease

The ε4 allele occurs in approximately 14% of the general German population, but its frequency decreases significantly with age, dropping to about 5% in centenarians [21]. Thus, it is a major mortality factor in the elderly, supposedly via its predisposition to both Alzheimer and cardiovascular diseases [22,23].

The physiological and molecular mechanisms of the associations between apoE genotype and fasting and non-fasting lipid levels are complex, and have been reviewed previously (see [7]). In general apoE4 carriers have a tendency for 5-10% higher fasting total cholesterol, LDL-cholesterol and triglyceride levels relative to homozygote ε3/ε3 [7,24]. This tendency towards higher lipid levels is likely to be in part responsible for the 40-50% greater cardiovascular disease (CVD) risk in ε4 carriers [25,26]; however additional mechanisms such as differences in oxidative status and chronic inflammation between apoE3 and apoE4 carriers may contribute to the observed differences in the CVD risk [8].

Although the molecular basis of the pathology is poorly understood, and likely to be in part due to apoE genotype associated differences in brain lipid metabolism, an apoE4 genotype has been highly consistently associated with the risk of an age-related loss of cognitive function, in an allele dose fashion. In a meta-analysis of the AlzGene database, Bertram and colleagues [27] reported odds ratios (OR) of Alzheimer's disease (AD) in Caucasians of 4.3 (95% CI 3.3-5.5) and 15.6 (95% CI 10.9-22.5) in ε3/ε4 and ε4/ε4 subgroups relative to the common ε3/ε3 genotype, which was consistent with the findings of an earlier meta-analysis [28].

### Additional roles of apoE as a modulator of immune function

Lipoproteins, including apoE-containing lipoproteins, have the ability to modulate key elements of the immune response by either inhibiting or stimulating antigen and mitogen induced T-lymphocyte activation as well as proliferation [29]. ApoE interacts with signals from multiple mitogens including transferrin and interleukin 2 (IL-2). Proliferation of both CD4 and CD8 lymphocytes is suppressed by apoE reducing the production of biologically active IL-2 [30]. In association with its immunomodulatory properties [31-33], apoE has an impact on the pathology of infectious diseases. Furthermore, recent research has demonstrated isoform-specific susceptibility to several viral infections [2,8].

The present review article summarises the impact of the ε4 allele on the susceptibility to specific infectious virus diseases, namely hepatitis C, human immunodeficiency virus (HIV) disease and herpes simplex. Furthermore, ε4 allele frequencies in different populations around the world are described. Potential mechanisms underlying the protective or adverse effects of the apoE isoforms in these specific virus diseases are given. We also discuss the prevalence of the ε4 allele in different global populations as well as the relationship between a higher frequency in certain regions and a possible protective effect of the apoE4 isoform on commonly encountered viral infections.

## ApoE4 allele frequencies in different populations

The frequencies of the apoE alleles vary between different ethnicities. The ε3 allele is usually the most prevalent, present in 50-90% of individuals, whereas ε2 has the lowest frequency at 0-15%, and is even absent in some native populations. The ε4 allelic variant occurs at a frequency of 5-30% [34].

In Europe the ε4 allele has a lower frequency in the southern rather than in the northern countries with continuously increasing frequencies with increasing latitude. The geographical decline from ~20% in Finland [35] to ~8% in Italy [36] and Greece [37] is likely to contribute to the north-south gradient in the prevalence of cardiovascular diseases [7,38,39]. In addition, ε4 tends to be less frequent in Eastern Europe. For example, ~11% of the Polish population carry an ε4 allele [40], while the proportion is ~14-15% in Germany and the Netherlands [41,42]. However, differences in ε4 allele frequency are also evident within a population depending on ethnicity or geographical position. For instance, 8.5% of the individuals living in central Italy carry an ε4 allele, while the proportion is slightly lower in southern Italy with 8.3% but only 5.3% in Sardinia, an island approximately 250 km west of the Italian mainland [36].

Remarkably, the highest frequencies of ε4 are found in indigenous populations and those with a recent history of hunter gatherer lifestyle whereas long-established agricultural communities (such as those in the Middle East, southern Europe, in Southeast Asia and Central America) show the lowest frequencies [38]. African populations exhibit a two to three times higher frequency than populations from Central Europe. About 37% of native Africans living in South Africa carry the ε4 allele [43]. The highest apoE4 frequency among populations of northern Europe and North America was found in Inuit from the eastern part of Greenland with 23% ε4 carriers [34]. The proportion of ε4 observed in Aborigines (26%) is twice as high as that in Australians of European descent (11%) [44,45]. Aceves *et al*. [46] observed a heterogeneous ε4 frequency in Mexicans, depending on the area's status of urbanisation as well as on the proportion of European gene flow, particularly that from Spanish colonisers. In Guadalajara, the second largest metropolitan area of Mexico with a relatively high proportion of Spanish descent habitants, the ε4 frequency was lowest at 8.4%, increased in the adjoining state Nayarit and reached its highest frequency of 28% in the Huichol Indians, a native Mexican population [46].

## Hepatitis C

Hepatitis C virus (HCV) infection is a major global health problem with more than 170 million infected people worldwide [47]. The consequences of HCV infection vary, as some newly infected individuals develop minor or no liver damage and recover spontaneously by clearing the virus [48], whereas approximately 85% suffer from progressive chronic hepatitis C, which is the leading cause of cirrhosis and hepatocellular carcinoma in the Western world [49,50]. Approximately 20-25% of HCV patients develop liver cirrhosis [51]. Accepted host factors for chronic HCV infection and progression of fibrosis and cirrhosis are older age at infection, male gender, excessive consumption of alcohol, insulin resistance, and the duration of infection [52,53]. In addition, a relationship between HCV and lipid metabolism has recently been suggested, since serum cholesterol levels are lower in patients with chronic HCV than in appropriately matched controls [54].

After infection, HCV circulates in the blood in a complex with lipoproteins. In the initial phase of infection, HCV virions are mainly associated with LDL, while later in the course of infection they are detectable in an assembly with HDL [39,55]. Attachment to hepatocytes is facilitated by binding of HCV envelope glycoprotein E2 to HSPG [56] with entry site of HCV into hepatocytes mainly via the LDL-R [57]. Due to the fact that both HCV and apoE bind to LDL-R and scavenger receptor [53] and that the apoE genotype affects receptor binding including a decreased number of LDL-R in apoE4 [58], it may be hypothesised that the apoE genotype impacts on HCV pathology. An overview of studies concerning the impact of apoE isoforms on HCV infection, outcome and course of disease in humans is presented in table 2.

**Table 2:** Effects of an apoE4 genotype on hepatitis C virus (HCV) infection and outcome in humans

Reference	Subjects and profile	Parameter	Outcome
Wozniak *et al*. 2002 [48]	156 HCV patients: 111 chronically infected and 45 with cleared infection, and 104 non-HCV infected patients	- risk of HCV infection - risk of severe liver disease caused by chronic HCV infection - risk of non-HCV associated liver diseases and extent of disease	ε2 = ε3 = ε4 non-ε4 > ε4 ε2 = ε3 = ε4
Mueller *et al*. 2003 [60]	506 chronically infected HCV patients	response to antiviral treatment of HCV infection	non-ε4 > ε4
Price *et al*. 2006 [53]	420 Caucasian HCV patients: 312 chronically infected and 108 with cleared infection, and 288 healthy controls	risk of chronic HCV infection	ε3 > ε4 > ε2
Mueller *et al*. 2007 [59]	701 HCV patients chronically infected, 523 healthy controls, and 283 patients with non-HCV associated liver diseases	- risk of chronic HCV infection - risk of severe non-HCV associated liver disease	non-ε4 > ε4 ε4 = non- ε4

HCV: hepatitis C virus

Wozniak *et al*. [48] investigated the association between apoE genotype and outcome of a HCV infection among HCV patients with chronic infection (defined as having detectable HCV RNA in the serum) or cleared infection (defined as presence of anti-HCV antibodies but absence of viral RNA in the serum). The apoE allele frequencies in the HCV infected groups were similar to those in the control group, with no differences between the HCV RNA-positive group and the negative individuals. In contrast, Price *et al*. [53] found a significantly lower number of subjects homozygous for ε2 in Caucasian hepatitis C patients compared to healthy controls, indicating a protective effect against HCV infection of the apoE ε2/ε2 genotype [53]. In addition, risk of developing a chronic HCV infection was highest in the ε3/ε3 genotype with odds ratio of 0.39 and 0.59 for the ε2 and ε4 allele, respectively, supporting the hypothesis that these genotypes are favouring viral clearance [53].

The association between severity of HCV-induced liver damage and apoE genotype was also examined revealing significantly lower ε4 allele frequencies in patients with severe versus mild inflammation and fibrosis [48] and a reduced risk of viral persistence [53] and chronic infection [59] in apoE4 carriers, suggesting that apoE4 protects HCV infected patients from developing severe liver disease. However, no significant impact of apoE genotype on non-HCV associated liver disease has been established [48,59].

Interestingly, response to antiviral treatment was higher in chronic HCV-patients with an ε3/ε3 genotype than with an ε3/ε4 and ε4/ε4 genotype [60].

### Possible mechanisms of the impact of apoE4 on hepatitis C

**Figure 1:**
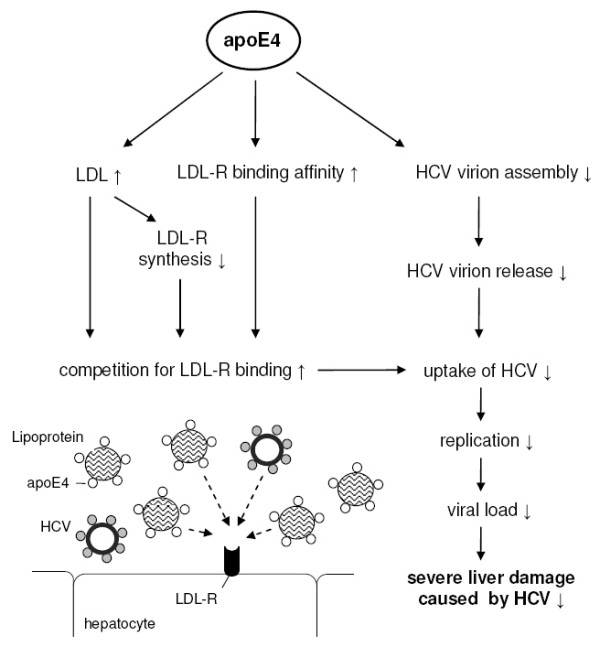
**Potential mechanisms of the impact of apoE4 on viral treatment response, fibrosis progression in recurrent HCV infection as well as protection against HCV associated severe liver damage**. LDL: low density lipoprotein, LDL-R: low density lipoprotein receptor, HCV: hepatitis C virus

Although several studies emphasise an impact of the apoE genotype on the course of HCV infection as well as on the outcome of HCV related liver damage, the underlying mechanisms of apoE-HCV-interactions remain to be elucidated. A summary of proposed mechanisms is given in figure 1.

Chang *et al*. [49] investigated the properties of HCV virions and the role of apoE in HCV infectivity and virus production in an *in vitro *study using human hepatoma cell lines that stably produce infectious HCV. Low-density HCV virions (1.08 to 1.12 g/ml) were found to be infectious whereas the majority of HCV RNA-containing particles emerging in higher-density fractions (1.15 to 1.21 g/ml) were poorly or not infectious. Additionally, infectious low-density HCV virions were rich in apoE, and levels of apoE and HCV RNA in infectious fractions were positively correlated. A short interfering RNA (siRNA)-mediated knockdown of apoE expression reduced apoE secretion and caused an apparent dose-dependent suppression of HCV virion production in the cells. However, the amount of HCV RNA-containing particles was not significantly affected by an apoE overexpression indicating that the level of apoE expression is not a limiting factor for HCV replication. Chang *et al*. [49] suggested that apoE knockdown-associated reduction of HCV RNA-containing particles was not related to an inhibition of HCV release but was more likely due to the blockage of apoE dependent HCV virion assembly. Taken together these results demonstrate that *in vitro *apoE is required for the production of low-density HCV particles as well as for their infectivity. However, mechanisms of apoE genotype-specific effects on HCV life cycle remain uncertain [49]. Since HCV particles are supposed to enter target cells via LDL-R [57] and thus, compete with apoE containing lipoproteins for receptor binding [1], it was hypothesised that the already established decreased LDL-R expression in ε4 vs. ε3 carriers [58] could diminish hepatic HCV uptake and spread between hepatocytes [48].

In summary apoE seems to be involved in the HCV life cycle. Although the mechanisms are yet poorly understood, the apoE4 isoform appears to have a HCV-specific protective effect on liver disease with available data indicating that the outcome of chronic HCV infection is better among ε4 carriers due to slower fibrosis progression. Limited evidence indicates that the ε4 allele may also be associated with poor viral treatment response.

## HIV infection and HIV-associated dementia

Infection with HIV and progression to the acquired immunodeficiency syndrome (AIDS) is a growing global health problem. At the end of 2007, the Joint United Nations Programme on HIV/AIDS estimated that 33.2 million people were living with HIV, 95% of them in developing countries [61].

Target cells of HIV are macrophages and brain microglia, in particular CD4^+ ^T-lymphocyte cells [62]. These cells are infected by HSPG mediated virus attachment [63] followed by binding of HIV envelope glycoproteins to the CD4 receptor. The resulting conformational change allows the envelope glycoproteins to bind to one of the two possible co-receptors CCR5 and CXCR4 [62]. In preparation of cell entry, HIV fuses with the target cell membrane, which requires cholesterol in HIV particles and lipid rafts (membrane microdomains enriched in certain lipids, cholesterol and proteins) [64,65]. The infected cells synthesise HIV proteins including the HIV transactivator protein (Tat) that activates viral and cellular gene expression [66]. Newly assembled virus particles are transported to the cell surface and released by exocytotic fusion with the plasma membrane for further cell infection [67].

The acute phase of HIV infection is characterised by extensive and rapid loss of CD4^+ ^cells due to apoptosis that persists into the chronic phase of infection. Plasma levels of CD4^+ ^cells are partially reconstituted after the first infection period, but then decrease continuously. Counting CD4^+ ^cells is therefore a key measure to assess progression of HIV disease [68]. Antiretroviral therapy is currently the best known way to protect mucosal tissue and to prevent microbial translocation and chronic activation of the immune system. It reduces the plasma levels of HIV RNA and induces a greater reconstitution of CD4^+ ^cells [68]. A low CD4^+ ^cell count is an independent risk factor for HIV-associated dementia [69] which is developed by approximately one-third of HIV infected adults. HIV-associated dementia is accompanied by microglial cell activation, astrocytosis, decreased synaptic and dendritic density and selective neuronal loss [70]. Symptomatic parallels between HIV-associated dementia and AD have been previously reported [69,71]. HIV infection of microglia, neurons and astrocytes in associated with stimulation of tumor necrosis factor α (TNF-α) and inducible nitric oxide synthase (iNOS) expression in the neighbouring uninfected cells [72], which have also been observed in AD [69]. *In vitro*, TNF-α substantially enhances the transcription of HIV in chronically infected mononuclear cells [73-75]. The pathogenesis of HIV and AIDS is therefore directly connected with the activation state of the host immune system [76].

Given that cholesterol is a crucial component of the HIV envelope and essential for viral entry and assembly [64,77,78] and that apoE is essential for cholesterol transport [10], along with being a mediator of brain inflammatory process [79], it may be hypothesised that the apoE genotype influences HIV-induced effects on neurological functions. Table 3 summarises studies on the effect of apoE4 on HIV pathogenesis and related disorders in humans.

**Table 3:** Effects of apoE4 genotype on HIV course of disease and HIV-associated dementia in humans

Reference	Subjects and profile	Parameter	Outcome
Corder *et al*. 1998 [69]	44 HIV infected patients	- risk of HIV-associated dementia - risk of peripheral neuropathy in HIV infection	ε4 > non-ε4 ε4 > non-ε4
Dunlop *et al*. 1997 [81]	132 AIDS patients and postmortem samples of hippocampus	risk of HIV-associated dementia and encephalitis	ε2 = ε3 = ε4
Burt *et al*. 2008 [63]	1,267 HIV-positive patients and 1,132 healthy controls	- risk of acquiring HIV infection - acceleration of HIV disease progression - increase of steady-state viral load - risk of HIV-associated dementia	ε2 = ε3 = ε4 ε4/ε4 > ε3/non-ε3 and ε3/ε3 ε4/ε4 > ε4/non-ε4 > non-ε4/non-ε4 ε4 = non-ε4
Valcour *et al*. 2004 [80]	222 HIV-positive patients of the Hawaii Aging with HIV Cohort	- ε4 allele frequency - risk of HIV-associated dementia	younger cohort > older cohort ε4 > non-ε4 within the older cohort

AIDS: acquired immunodeficiency syndrome, HIV: human immunodeficiency virus

In a study by Corder *et al*. [69] HIV infected patients were evaluated every six months during a period of five years. Although non-significant, a tendency towards decreased CD4^+ ^cell count was evident in ε4 carriers. In addition, a highly significant difference of frequencies of neurological symptoms was evident with an incidence of 30% in ε4 carriers and 15% in non-ε4 carriers. Peripheral neuropathy was also more common among individuals carrying ε4 [69].

An acceleration of the HIV disease course by apoE4 has also been observed in the Hawaii Aging with HIV Cohort. Those HIV patients who possessed at least one ε4 allele tended to have a faster self-reported HIV progression with 8.3 compared to 10.5 years. However, CD4^+ ^cell count as an indicator of disease progression as well as plasma HIV RNA and intracellular HIV DNA levels were not affected by apoE4 [71]. Further classification of the cohort revealed that the ε4 allele was more frequent in the younger group (<40 years, 37%), than in the older group (>50 years, 23%) indicating that ε4 may influence survival. In contrast to the younger individuals, in the older cohort an apoE4 genotype was associated with an increased risk of HIV-associated dementia after controlling for age and diabetes status [80]. These findings highlight the possibility of an impact of age on the ability of apoE genotype to module the risk of developing HIV-associated dementia [71].

However further larger studies have failed to confirm this impact of apoE genotype on HIV-associated dementia prevalence [63,81]. Dunlop *et al*. [81] did not observe any differences in allele distribution among AIDS patients with clinical signs of dementia or histological signs of encephalitis relative to patients free of these clinical symptoms. The impact of a homozygous ε4/ε4 genotype was not determinable due to the low presence among the study participants [81]. Consistent with the Dunlop findings Burt *et al*. [63] did not observe any significant impact of genotype on the risk of acquiring HIV infection and HIV-associated dementia. However there was some evidence to suggest that apoE genotype influenced the course of infection, with homozygosity for the ε4 allele associated with an accelerated course of disease. The impact of ε4/ε4 genotype on disease progression was not influenced by CD4^+ ^cell count. In contrast, the effect of ε4/ε4 genotype on the course of HIV was minimised after adjustment for the steady-state viral load, indicating that the extent of viral replication is affected by apoE genotype.

Furthermore, Burt *et al*. demonstrated in an *in vitro *model that the cell entry of HIV strains is apoE isoform dependent with a significantly higher cell infection rate in the presence of apoE4 compared with apoE3, which may in part explain the higher infection rate in ε4 carriers [63].

In summary, available evidence indicates that the ε4 allele has detrimental effects on the course of HIV infections. It has been associated with higher steady-state viral load and faster disease progression due to accelerated virus entry in ε4 carriers. However, CD4^+ ^cell count does not appear to be significantly affected by the apoE genotype. The correlation between apoE4 and HIV-associated dementia is possibly age dependent, but remains controversial and needs to be clarified by further studies.

### Possible mechanisms of the impact of apoE4 on HIV infection and HIV-associated dementia

Although the risk of acquiring HIV infection is independent of the apoE allelic variant, carriage of ε4 is associated with an increased steady-state viral load and a faster progression of HIV disease. Furthermore, an increased cell entry of HIV *in vitro *strains was discovered in the presence of apoE4 compared to the presence of apoE3 [63].

**Figure 2:**
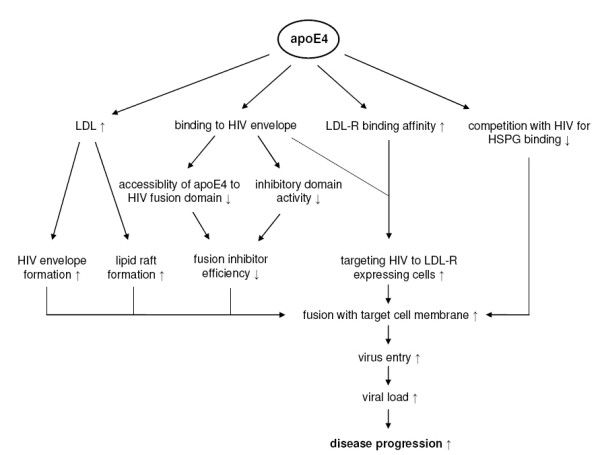
**Potential mechanisms for apoE4 mediated faster progression of HIV disease**. LDL: low density lipoprotein, LDL-R: low density lipoprotein receptor, HIV: human immunodeficiency virus, HSPG: heparin sulphate proteoglycans.

One possible underlying mechanism may be an impact of apoE genotype on LDL-R and HSPG binding activities which would directly impact on cellular HIV particle uptake (figure 2). ApoE found on the virus' envelope may facilitate virus entry by targeting HIV virions to the LDL-R expressing cells [82]. ApoE4's modestly higher receptor binding affinity may promote virus contact to target cells and hence increases HIV cell entry and as a consequence disease progression.

Dobson *et al*. demonstrated in an *in vitro *experiment that short apoE fragments containing the heparin-binding domain, namely residues 142 to 147, have antiviral activity against HIV infection. This effect possibly results from blockade of HIV attachment to HSPG (figure 2) [83]. The observation of lower HSPG mediated VLDL uptake of apoE4 versus apoE2 and apoE3 containing lipoproteins in a variety of cells cell lines [84] suggests that in apoE4 individuals lower competition is likely to be associated with accelerated HSPG mediated HIV cell entry.

In addition, the amphipathic helical domain of apolipoproteins shows homology with the viral fusogenic domains [63,83,85] and therefore Burt *et al*. [63] proposed that the amphipathic helical domain of apoE may act as a HIV fusion inhibitor by binding to the HIV membrane protein gp41 and blocking either the formation or the function of the HIV N-terminal fusogenic domain. Based on the observation that apoE4, compared to apoE3, enhances HIV fusion and cell entry, apoE4 seems to be a less efficient fusion inhibitor [63]. The differential antiviral activity of apoE isoforms may be due to either these structural characteristics that make the amphipathic helical domain of apoE4 less accessible to HIV fusion domains or to isoform-specific changes in inhibitory domain activity [63] (figure 2).

Cholesterol is a crucial component of the HIV envelope as well as of cholesterol rich lipid rafts which are essential for both, HIV entry and release [64,77,78]. Consequently, depletion of cholesterol from either the cell membrane or the HIV envelope results in a loss of infectivity [64,77]. ApoE4 genotype is associated with higher LDL levels and might therefore support an accumulation of plasma membrane raft-associated cholesterol and an increase of lipid raft formation, resulting in an enhancement of HIV infection cycle including HIV fusion and cell entry as well as assembly of HIV virions and its release [63]. In addition, the increased LDL levels in ε4 carriers may promote the formation of HIV envelopes and therewith of HIV virions, presumably resulting in higher viral load and faster disease progression as summarised in figure 2.

The validity and relative importance of the above mentioned mechanisms proposed to underlie the impact of apoE genotype on HIV cell entry and viral load requires further investigation.

The neurotoxic protein Tat has been proposed to be involved in the development of HIV-associated dementia [66,70]. Tat is actively released mainly from HIV infected macrophages, astrocytes and microglia and is taken up by uninfected cells [66,86-90]. It is supposed to interact with neuronal membranes inducing oxidative stress. A higher Tat-induced oxidative damage was evident in wild-type relative to apoE-knockout mice attributed to a competition between apoE and Tat for LRP-R uptake [66]. In the presence of murine apoE, the contact time of Tat with cell membranes is prolonged, increasing the degree of Tat-induced oxidative damage. In cortical neurones Tat-induced toxicity was lower in presence of lipid-associated human apoE3 as compared to apoE4 indicating less oxidative damage in membrane lipids and proteins [66].

**Figure 3:**
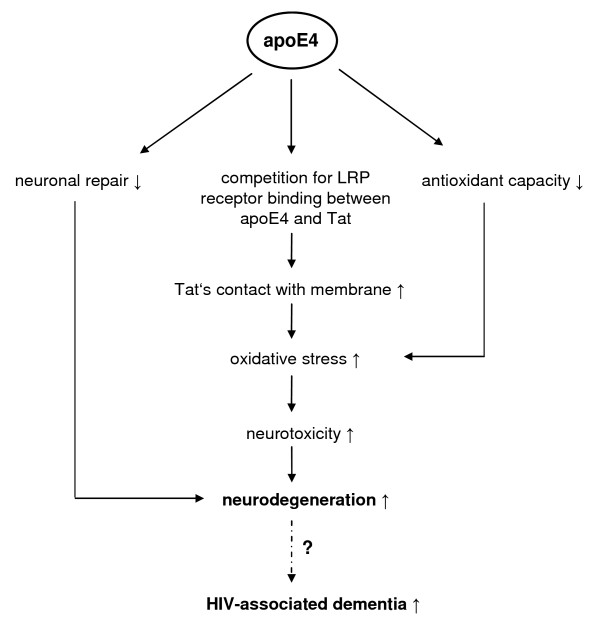
**Possible mechanisms of the impact of apoE4 on increased neurodegeneration and HIV-associated dementia**. Tat: transactivator protein, LRP: low density lipoprotein receptor-related protein, HIV: human immunodeficiency virus.

Figure 3 schematically summarises possible mechanisms underlying apoE genotype-HIV-associated dementia-interactions.

## Herpes simplex

Herpes simplex is a neurotropic infectious disease caused by a herpes simplex virus (HSV) which is an enveloped double stranded DNA virus [91] classified into two subspecies. Herpes simplex virus type 1 (HSV-1) contributes to the pathogenesis of numerous clinical conditions in humans with herpes labialis, also known as cold scores or fever blisters, being the symptom most often recognised [1], while herpes simplex virus type 2 (HSV-2) usually causes genital infections [92]. Seroprevalences of HSV-1 and HSV-2 infections increase with age. HSV-1 is spread more widely than HSV-2 [92] and one of the most commonly encountered pathogens with a prevalence of 40-50% among adolescents and 60-90% among adult populations [93].

HSV-1 DNA is detectable in brain regions mainly affected in AD [94]. Though HSV-1 has been described as reducing overall synthesis of most host cell proteins, it has been shown to trigger cellular β-amyloid production and accumulation, which is a major hallmark of AD [95]. Interestingly, β-amyloid peptide and glycoprotein B of HSV-1 viral envelopes show significant amino acid sequence homology [96].

There are few studies investigating a potential association between HSV-1 infection, apoE4 genotype and development of AD with inconsistent outcomes (summarised in table 4).

**Table 4:** Effects of apoE4 genotype on herpes simplex virus (HSV)-1 infection and outcome in humans

Reference	Subjects and profile	Parameter	Outcome
Itzhaki *et al*. 1997 [98]	46 Caucasian AD patients and 44 Caucasian non-AD elderly controls	risk of AD	highest in ε4 carriers positive for HSV-1
	40 Caucasian cold-score sufferers and 33 age-matched Caucasian healthy controls	risk of HSV-1-induced cold-scores	ε4 > non-ε4
Itabashi *et al*. 1997 [99]	46 elderly AD patients and 23 age-matched controls without confirmed neuropsychiatric disease	HSV and risk of AD	latent HSV infection alone is not an independent risk factor for AD, the combination of ε4 and HSV infection increases the risk
Beffert *et al*. 1998 [97]	73 elderly AD patients and 33 non-AD controls	- susceptibility to HSV-1 infection in brain - risk of AD	ε4 = non-ε4 no effect of HSV-1 and ε4 combined
Letenneur *et al*. 2008 [100]	512 French individuals in a population based cohort study	apoE genotype and HSV immunoglobulin status as risk factors for AD	no interactions between apoE status and HSV immunoglobulin status on risk of AD

HSV: herpes simplex virus, AD: Alzheimer's disease, CNS: central nervous system

The susceptibility to HSV-1 infection was not affected by apoE genotype [97]. In an elderly cohort, apoE ε4 allele frequency was found to be highest among HSV-1-positive AD patients compared to HSV-1-negative AD patients and to non-AD patients indicating a several times higher AD risk for subjects carrying ε4 allele with HSV-1 infection than for non-ε4 carriers [98]. Thus, the HSV-1 infection alone shows no effect, but in combination with the apoE4 genotype the risk of AD increases apparently in the elderly [99]. Increasing age may facilitate viral entry into the CNS by declined immune or barrier function since HSV-1 was not detected in brains of younger people [98]. In addition to the increased susceptibility of the central nervous system the apoE4 genotype was also reported to increase the risk of HSV-1 induced cold scores in the peripheral nervous tissue [98].

In contrast in a prospective population based study of 512 non-demented elderly subjects a significant association between primary HSV-1 infection or reactivation (IgM-positive status) and development of AD was found. Over the period of 14 years 99 subjects developed dementia including 77 cases of AD. However, no interactions between the apoE genotype and IgM-positive status on the risk of AD could be observed, although the relatively small number of ε4 carriers with HSV-1 infection has to be considered [100]. Nevertheless, in a previous study the combination of HSV-1 infection and apoE4 genotype did also not reveal any significant effect on the prevalence of AD [97].

Studies in transgenic apoE mice provide further evidence of an interaction between the apoE genotype and HSV-1 infection. Burgos *et al*. [101] analysed the involvement of different apoE isoforms in HSV-1 access to the CNS during the acute phase of infection. Thus, 14-week-old female apoE3 and apoE4 transgenic mice were inoculated with HSV-1 and the infection status of several tissues was analysed six days after inoculation. ApoE isoform dependent differences in the viral load in the CNS and certain brain regions were observed. ApoE4 transgenic mice had significantly higher HSV-1 levels in the spinal cord and in all regions of the brain. HSV-1 levels in the cortex, cerebellum and ventricles of apoE3 transgenic mice were undetectable, while apoE4 transgenic mice showed significantly higher levels of HSV-1 in these brain regions. However, no significant differences were detected in the blood and the adrenal glands. These data show that apoE4 facilitates migration of HSV-1 from the adrenal gland to the brain ten times more efficiently than apoE3 [101].

In a further study, the influence of the apoE profile on levels of latent HSV-1 DNA was investigated in the same rodent model [94]. Since HSV-1 is present in a latent state four weeks after inoculation, viral DNA concentration was determined five weeks after infection, when latency was assured. The highest virus loads were found in the nervous system, including spinal cord, brain and trigeminal ganglia, and in the blood possibly derived from the trigeminal ganglia. Therefore, it has been suggested that a high viral load is accompanied with establishment of HSV-1 latency in the respective tissues. Wild-type mice showed significantly higher concentrations of HSV-1 than apoE-knockout mice in the nervous tissue, adrenal glands and ovaries but similar concentrations in the blood. In comparison to apoE3 mice, apoE4 mice had higher levels of latent HSV-1 DNA, except for the adrenal glands and the blood. In the apoE4 mice HSV-1 was detected in all brain regions analysed, while in apoE3 mice HSV-1 was only detectable in the midbrain. Latent HSV-1 DNA levels in the nervous system were twelve times higher in the presence of apoE4 compared to apoE3 [94]. Burgos *et al*. [94] suggested that apoE4 may increase the risk of developing AD by increasing latent HSV-1 viral load in the nervous system. The ability to cause damage may depend on the exact location, the immune status of the host, and the amount of virus (in which apoE genotype status could play an important role) [94].

In conclusion, current literature indicates that apoE4 intensifies the susceptibility for HSV-1 related herpes labialis as well as the neuroinvasiveness of HSV-1 compared to other apoE variants and that the combination of apoE4 and HSV-1 may lead to a higher risk of AD, than either factor in isolation. However inconsistencies exist in the literature, which is likely to be in large part due to the lack of the statistical power of the human studies conducted to date.

### Possible mechanisms of the impact of apoE4 on herpes simplex (Figure 4)

**Figure 4:**
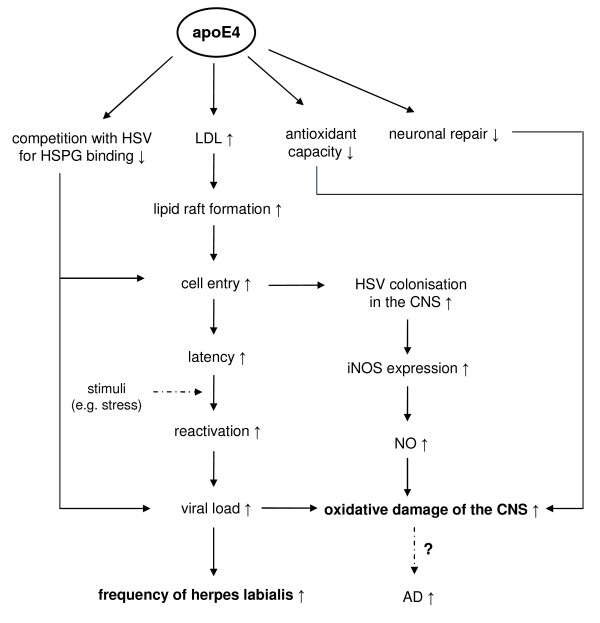
**Possible mechanisms of the adverse effects of apoE4 on the outcome of herpes labialis and the increased HSV-induced oxidative damage in the CNS that possibly increases the susceptibility to AD**. HSV: herpes simplex virus, HSPG: heparin sulphate proteoglycans, LDL: low density lipoprotein, iNOS: inducible nitric oxide synthase, NO: nitric oxide, CNS: central nervous system, AD: Alzheimer's disease.

Similar to HCV and HIV, HSV competes with apoE for receptor binding [1]. In addition HSV attachment to the target cells is facilitated by HSPG located on the cell's surface [102]. Therefore differences in apoE-HSPG associations are also likely to impact on the cellular HSV uptake as proposed by Itzhaki and Wozniak [1]. Furthermore, lipid rafts are involved in HSV entry as well [65]. As discussed for HIV, the higher levels of LDL present in ε4 carriers possibly support an accumulation of cholesterol in these specialised membrane domains and increases their formation [63]. This would enhance virus entry and therewith the chance for HSV to establish latency.

HSV-1 infection of the murine nervous system is associated with up-regulated iNOS expression as well as raised levels of pro-inflammatory cytokines [103,104] which undoubtedly play a central role in the neuronal injury associated with the viral infection. Given the observation of increased oxidative stress, inflammatory cytokine production and nitric oxide production associated with an apoE4 genotype [8,105-107] and the less efficient neuronal repair described in E4 carriers [69], this considerable overlap between the pathological effects of an apoE4 genotype and the neuronal impact of the HSV provides a likely mechanism to explain the observation of increased AD risk in E4 carriers associated with HSV infection.

## Conclusion

This review provides an overview of the so far available data on the involvement of the apoE genotype in the pathogenesis of specific virus diseases. It is conceivable that due to its beneficial effects in certain harmful infectious diseases, as demonstrated for hepatitis C, the ε4 allele may not have been eliminated by evolutionary selection. In such a way there exists a geographical north-south gradient in Italy with a higher number of HCV infections in the south of Italy [108] parallel to the inverse geographical gradient in apoE4 genotype frequency being lowest in southern Italy [36]. However, substantial studies are needed to correlate geographical differences and ethnic origins with the incidence of these virus infections.

The mechanisms of HCV, HIV and HSV infection bear resemblance to each other, since all three viruses compete with apoE for cell attachment and receptor binding. Therefore it would be interesting to investigate whether other types of viruses share the same mechanism and whether their cell entry is affected by the apoE genotype. This could contribute to our understanding of the apparent paradox that apoE4 competes efficiently with HCV for receptor binding but is less efficient in inhibiting the binding of HIV and HSV to the receptors.

Since the detailed mechanisms of the impact of apoE4 on the virus cell entry, the infection cycle and the virus-induced disorders are not completely understood, further experimental and clinical studies are needed.

The knowledge of the mechanisms by which apoE may influence the pathogenesis of infectious virus diseases may open up novel strategies to develop personalised antiviral treatments depending on the individual's apoE genotype.

## Competing interests

The authors declare that they have no competing interests.

## Authors' contributions

IK, AMM, PH, AN and GR wrote the manuscript. All authors contributed to the revision of the manuscript. IK prepared the figures of the manuscript.
